# Supplementary figures and images for: Understanding the impact of high-risk human papillomavirus on oropharyngeal squamous cell carcinomas in Taiwan: A retrospective cohort study
Source: PLoS One. 2021 Apr 23;16(4):e0250530. doi: 10.1371/journal.pone.0250530 (PMC8064583; doi:10.1371/journal.pone.0250530)

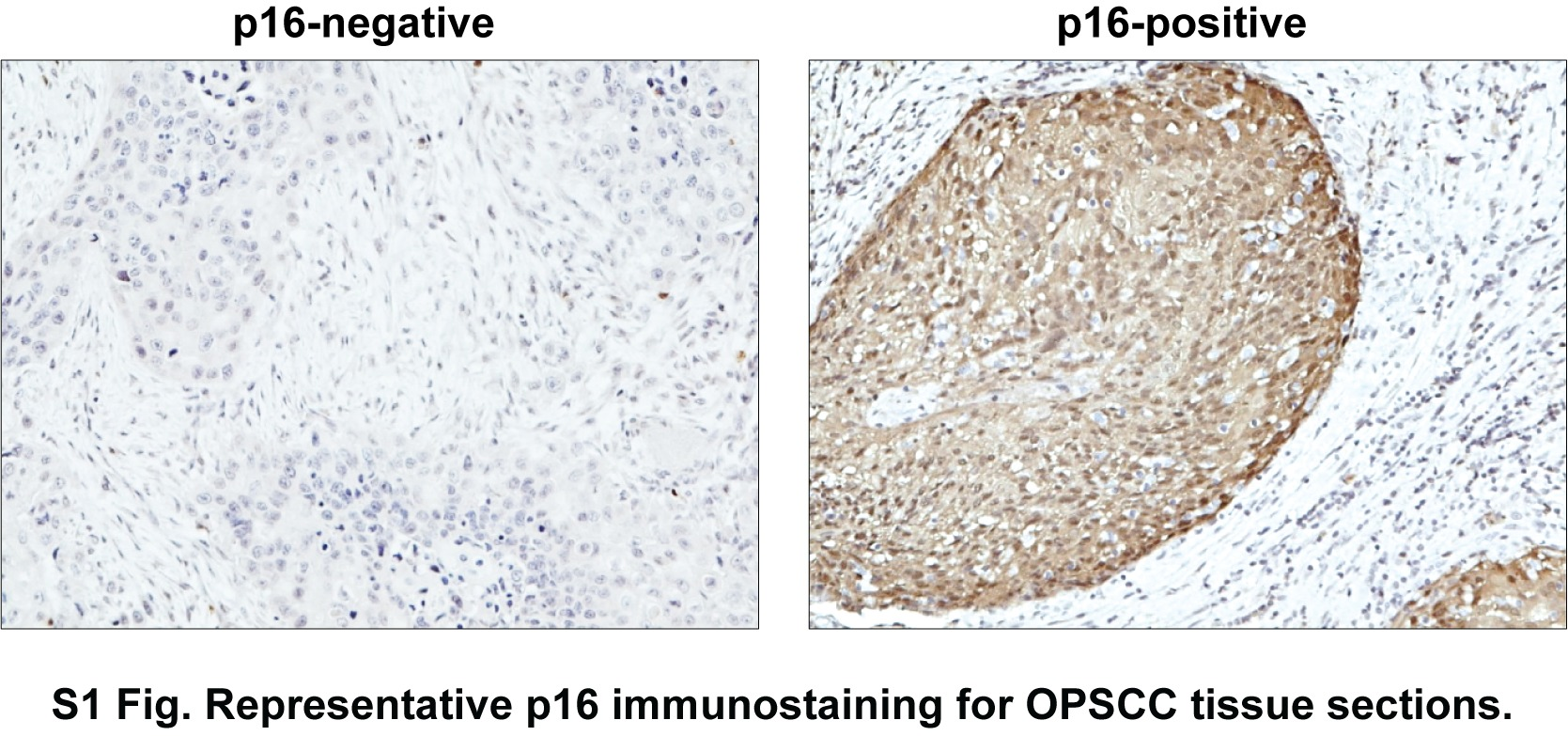

Supplement: S1 Fig — Specimens from example p16-negative (P0283) and p16-positive (P0267) tumors are displayed. p16 expression is observed as a brown nuclear and cytoplasmic coloration. Magnification, 200x. (TIF) [file pone.0250530.s002.tif]

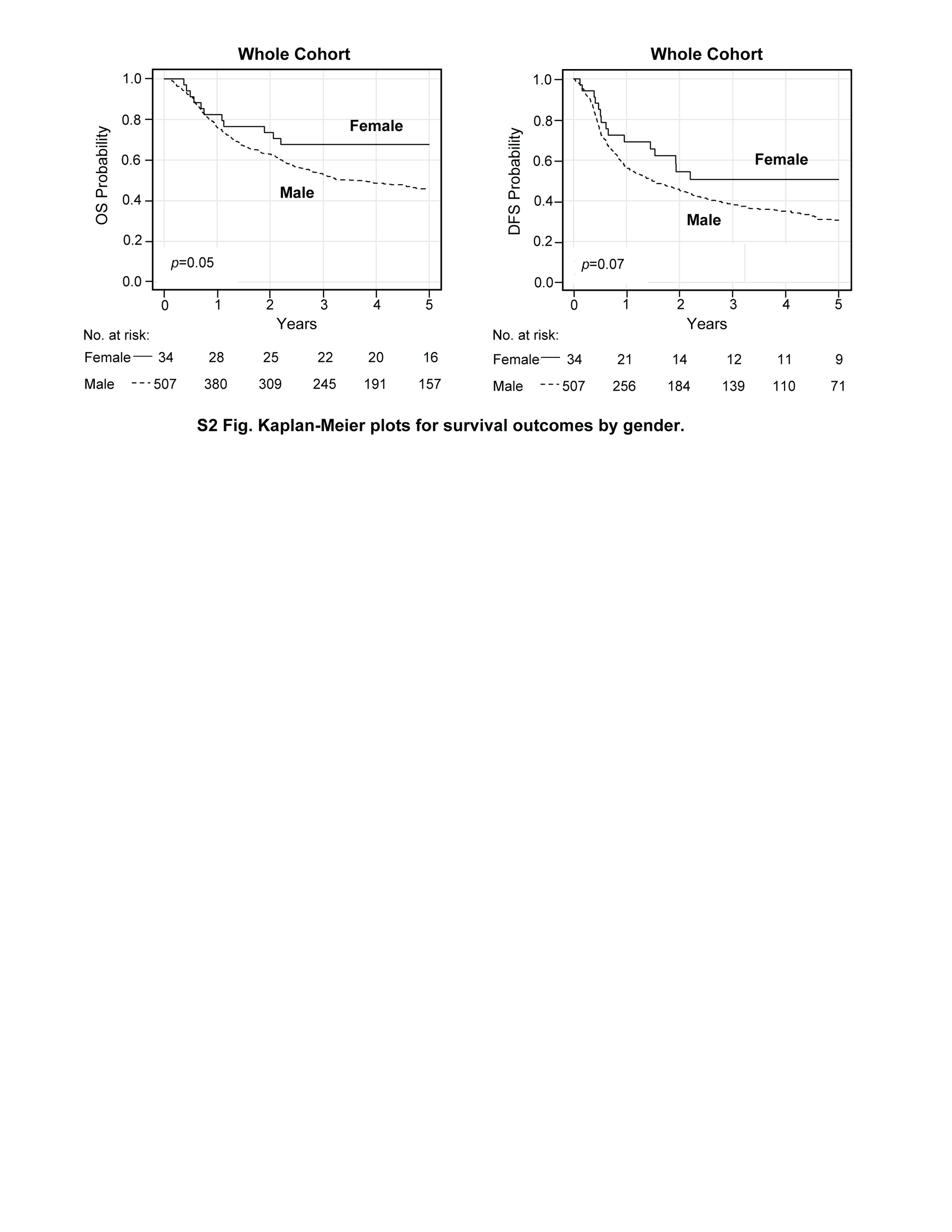

Supplement: S2 Fig — Up to 5-year overall survival (OS) and disease-free survival (DFS) outcomes were analyzed within the whole cohort by the Kaplan-Meier method and log-rank test (p-values). (TIF) [file pone.0250530.s003.tif]

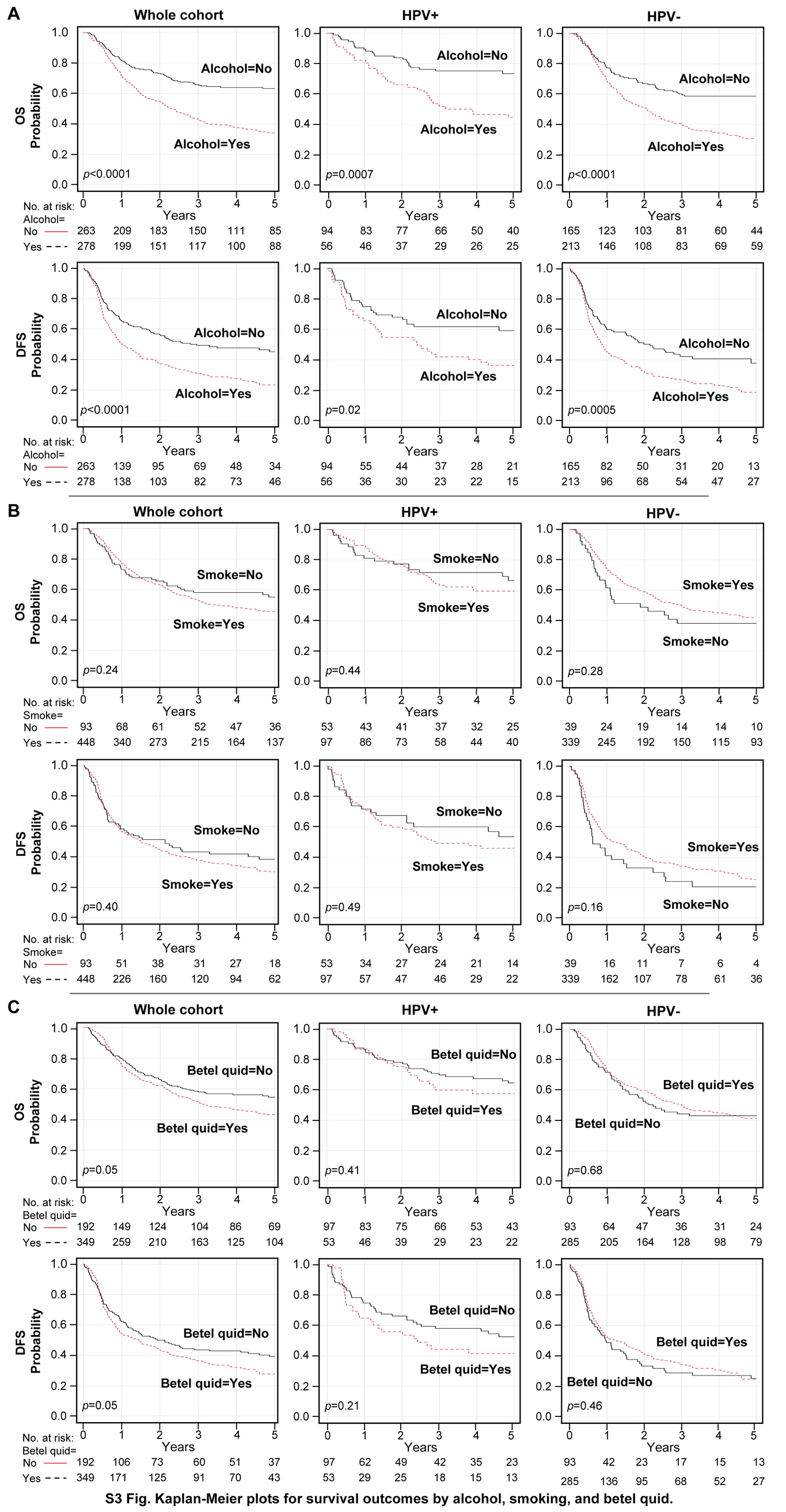

Supplement: S3 Fig — (A-C) Comparison of prognostic outcomes of (A) alcohol, (B) smoke, and (C) betel quid between the whole cohort and HPV risk groups. HPV positivity is defined as HPV DNA-positive and/or p16-positive. Up to 5-year overall survival (OS, top) and disease-free survival (DFS, bottom) probabilities were analyzed by the Kaplan-Meier method and log-rank test (p-values), as displayed for each risk group. HPV-, HPV-negative; HPV+, HPV-positive. (TIF) [file pone.0250530.s004.tif]

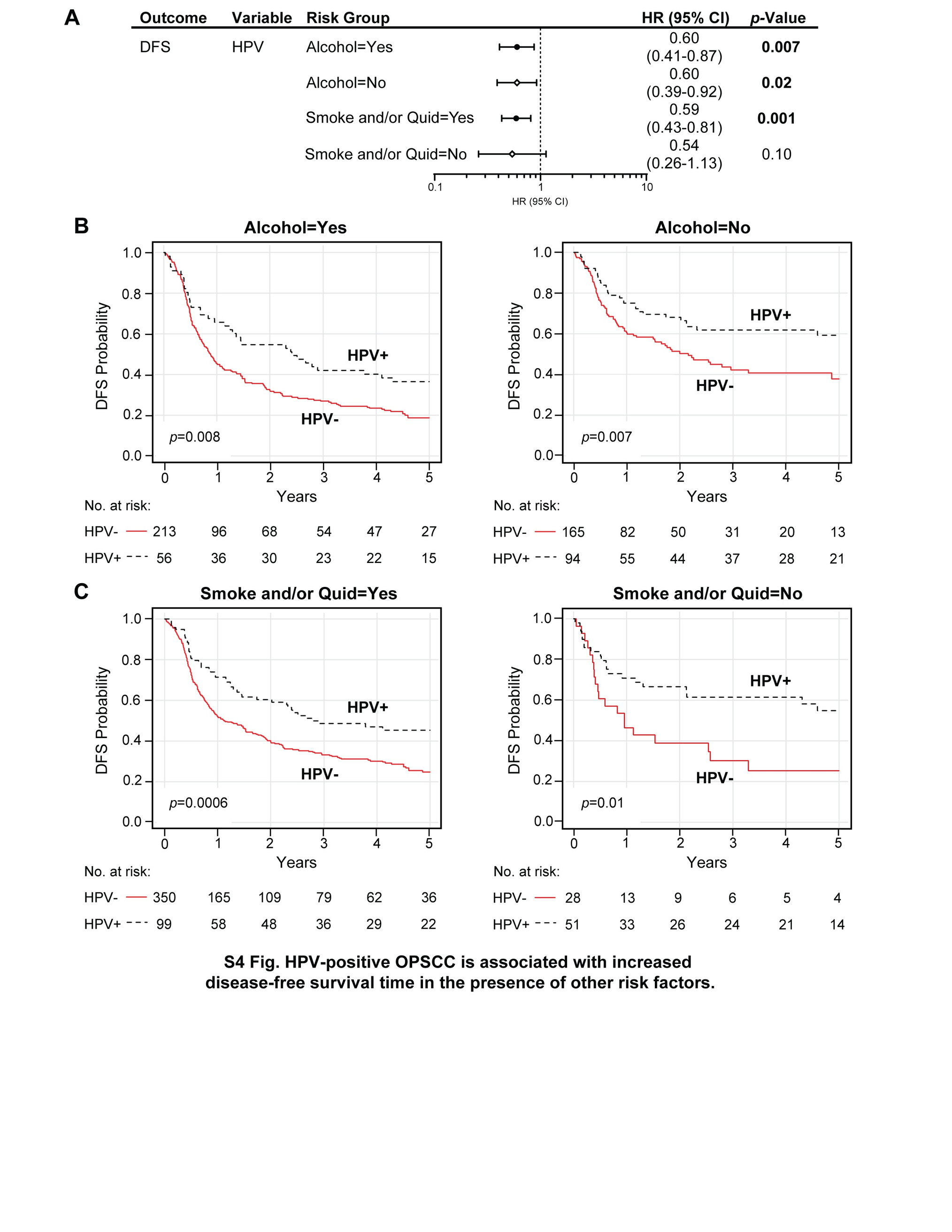

Supplement: S4 Fig — (A-C) Up to 5-year disease-free survival (DFS) prognostic outcome of the HPV variable within alcohol, and smoking and/or betel quid risk groups. HPV positivity is defined as HPV DNA-positive and/or p16-positive. The DFS smoking and betel quid variables were not analyzed individually due to low number of events. (A) Table includes the multivariable hazard probabilities analyzed using Cox survival models and hazard ratio (HR) estimations, adjusted for age, T- and N-stage, and which were visualized by forest plots. The complete analysis is found in S8 Table. (B-C) Kaplan-Meier survival analysis. Plots represent the DFS probabilities of cases stratified by HPV status within the (B) alcohol and (C) smoke and/or betel quid groups. Left, plots showing cases with alcohol or smoke and/or betel quid consumption. Right, plots showing cases without exposition to alcohol or smoke and/or betel quid. Log-rank analysis was used to compare the survival distributions (log-rank p-values are in the plots). HPV-, HPV-negative; HPV+, HPV-positive. (TIF) [file pone.0250530.s005.tif]
